# PET imaging shows no changes in TSPO brain density after IFN-α immune challenge in healthy human volunteers

**DOI:** 10.1038/s41398-020-0768-z

**Published:** 2020-03-09

**Authors:** M. A. Nettis, M. Veronese, N. Nikkheslat, N. Mariani, G. Lombardo, L. Sforzini, D. Enache, N. A. Harrison, F. E. Turkheimer, V. Mondelli, C. M. Pariante

**Affiliations:** 1 Institute of Psychiatry, Psychology and Neuroscience, King’s College London, Department of Psychological Medicine, London, UK; 2 National Institute for Health and Research Biomedical Research Centre at South London and Maudsley NHS Foundation Trust and King’s College London, London, UK; 3 Institute of Psychiatry, Psychology and Neuroscience, King’s College London Department of Neuroimaging, London, UK; 4 Universita’ degli Studi di Milano, Psychiatry Unit, Department of Biomedical and Clinical Sciences, Luigi Sacco Hospital, Milan, Italy; 5 Karolinska Institute, Department of Neurobiology, Care Sciences and Society, Division of Neurogeriatrics, Stockholm, Sweden; 6 Cardiff University Brain Research Imaging Centre (CUBRIC), Cardiff University, Cardiff, UK

**Keywords:** Molecular neuroscience, Diagnostic markers

## Abstract

Depression is associated with peripheral inflammation, but its link with brain microglial activity remains unclear. In seven healthy males, we used repeated translocator protein-Positron Emission Tomography (TSPO-PET) dynamic scans with [^11^C]PBR28 to image brain microglial activation before and 24 h after the immune challenge interferon (IFN)-α. We also investigated the association between changes in peripheral inflammation, changes in microglial activity, and changes in mood. IFN-α administration decreased [^11^C]PBR28 PET tissue volume of distribution (Vt) across the brain (−20 ± 4%; t_6_ = 4.1, *p* = 0.01), but after correction for radioligand free-plasma fraction there were no longer any changes (+23 ± 31%; t = 0.1, *p* = 0.91). IFN-α increased serum IL-6 (1826 ± 513%, t_6_ = −7.5, *p* < 0.001), IL-7 (39 ± 12%, t_6_ = −3.6, *p* = 0.01), IL-10 (328 ± 48%, t_6_ = −12.8, *p* < 0.001), and IFN-γ (272 ± 64%, t_6_ = −7.0, *p* < 0.001) at 4–6 h, and increased serum TNF-α (49 ± 7.6%, t_6_ = −7.5, *p* < 0.001), IL-8 (39 ± 12%, t_6_ = −3.5, p = 0.013), and C-reactive protein (1320 ± 459%, t_6_ = −7.2, p < 0.001) at 24 h. IFN-α induced temporary mood changes and sickness symptoms after 4–6 h, measured as an increase in POMS-2 total mood score, confusion and fatigue, and a decrease in vigor and friendliness (all *p* ≤ 0.04). No association was found between changes in peripheral inflammation and changes in PET or mood measures. Our work suggests that brain TSPO-PET signal is highly dependent of inflammation-induced changes in ligand binding to plasma proteins. This limits its usefulness as a sensitive marker of neuroinflammation and consequently, data interpretation. Thus, our results can be interpreted as showing either that [^11^C]PBR28 is not sensitive enough under these conditions, or that there is simply no microglial activation in this model.

## Introduction

Microglial activation has been proposed as the core neuroinflammatory process in psychiatric disorders. Microglia are brain resident macrophages and the primary immune effector cells in the central nervous system. Previous preclinical and postmortem studies investigating the role of inflammation in depression have shown an association between depressive symptoms and greater microglial activity^1–4^, suggesting that the brain’s innate immune response might play a role in the pathophysiology of depression. In vivo, microglial activation can be measured with positron emission tomography (PET) radioligands targeting translocator protein (TSPO), a protein located on outer mitochondrial membranes in microglia, as TSPO appears to be overexpressed when microglial cells are activated during neuroinflammation^5^.

Despite some negative results^6^, the majority of TSPO-PET studies in patients with depression have reported increased TSPO binding compared with healthy controls^7–11^, suggesting increased microglial activation in these patients. These studies thus propose that the inflammatory response in depression involves not only the periphery^12,13^ but also the brain. Indeed, behavioural symptoms of depression might be induced by peripheral cytokines and immune cells acting on the brain to elicit a neuroinflammatory response^14,15^. However, no PET study so far has found a correlation between peripheral and central inflammatory markers in patients with depression, and so the mechanism underpinning these co-existing findings remains unclear^4^. Moreover, a recent meta-analysis from our team^4^ has highlighted the heterogeneity of these PET studies in terms of study design, methods, and selected sample. For example, studies using first generation radiotracers used the binding potential as endpoint, which is calculated by normalizing the activity in the target region with the activity of a region devoid of TSPO; since TSPO is ubiquitous, in this case normalization is achieved by a pseudo-reference region with a kinetic profile similar to the one measured in healthy controls^16^. The quantification of second generation tracers, instead, mostly uses the volume of distribution as endpoint, which is the estimated ratio at equilibrium between the activity in the target and the activity in plasma^16^.

Of note, there are also quite a lot of differences between these studies in depression. For example, and of particular relevance to the present study, only two studies^6,11^ corrected for the free-plasma fraction of the ligand, that is, how much of the ligand is available for brain tissue binding as it is not bound to plasma proteins, which is important given the evidence of TSPO ligand binding to peripheral inflammatory proteins^17^. Moreover, studies included depressed patients with different ages (including late-life depression^8^), symptoms severity, and medication status^18^, and only three examined drug-free depressed patients^9–11^.

Considering the intrinsic clinical heterogeneity of the depressed clinical population, studies in healthy volunteers are important to dissect the mechanisms linking peripheral and central inflammation. Experimental models in animals have demonstrated that microglial activation can be induced by a peripheral immune challenge; for example, many studies have used Escherichia coli lipopolysaccharide (LPS) as peripheral immune challenge to elicit microglial activation in rodents^19,20^. In line with this evidence, two TSPO-PET studies have investigated non-human primates following LPS administration, and both showed a significant increase in TSPO [^11^C]PBR28 binding in the brain^21,22^. Moreover, the study by Hannestad and colleagues also found correlations between peripheral cytokines levels and the TSPO binding. So far, only one PET study (also with [^11^C]PBR28) has used LPS in humans, in eight healthy males, finding increased TSPO binding (by 30–60%) after 3 h from the LPS injection, although TSPO binding did not correlate with peripheral inflammation nor with measures of mood^23^.

In the present study, we examine peripherally induced systemic inflammation and brain TSPO binding in healthy humans using interferon (IFN)-α^24,25^. IFN-α is a pro-inflammatory cytokine with antiviral, anticancer, and immunomodulatory effects, approved for treatment of cancer and chronic hepatitis C^26,27^. IFN-α induces a systemic immune response associated with sickness behaviour, and, when administered over weeks or months as a treatment for patients with cancer or hepatitis C, is associated with a diagnosis of major depression in up to 30–50% of patients, and is thus considered the most validated clinical model of inflammation-induced depression^28^. Supporting our choice of using this model, preclinical studies have shown that peripheral IFN-α treatment in mice induces activation of microglia, and that this activation is associated with depressive-like behaviour^29,30^.

Similarly to the aforementioned LPS study in humans^23^, here we measure microglial activation with [^11^C]PBR28 PET, before and 24 h after a single IFN-α administration, in seven healthy male subjects. Of note, second generation ligands, such as [^11^C]PBR28, show a higher signal-to-noise ratio compared with first generation ones, facilitating measurement of significant within-subjects changes with a smaller sample size^5^. However, their binding affinity depends on the rs6971 nucleotide polymorphism on the TSPO gene^31^, and the quantification of the brain signal is confounded by the presence of abundant TSPO in endothelial cells and by the high ligand affinity for plasma proteins^32^. Here we apply a quantification methodology that takes into account all these limitations—something which the previous LPS study did not do. The decision to have the second PET scan 24 h after the IFN-α administration was based on preclinical studies showing that in vitro IFN-α-stimulated microglia releases inflammatory cytokines after approximately 24 h^29^. We also measure the levels of peripheral cytokines and the transient changes in mood after IFN-α. Based on the evidence discussed above, we hypothesize that the IFN-α injection would induce an increase in brain TSPO binding, in peripheral inflammation and in depressive-like symptoms, with possible correlations between these three sets of variables.

## Materials and methods

### Subjects

Seven healthy males provided written informed consent and participated in the study. This study was approved by the Queen Square London Ethical committee, ref. 16/LO/1520. Participants were recruited through King’s College London internal e-mail, online platforms and public advertising. In order to determine eligibility, participants had a pre-screening phone call, followed by a screening visit. Their medical history was collected, ad a MINI Psychiatric scale administered. Eligible participants were non-smokers, drank no more than five alcohol drinks per week, had no history of significant medical illness and did not meet the criteria for any current or past psychiatric or substance-dependence diagnosis. Subjects were excluded if they had had an infection in the last month or had regularly used anti-inflammatory drugs. Subjects were also instructed to abstain from alcohol, anti-inflammatory medication and physical exercise for 72 h before the scans. During the screening visit, participants were genotyped for the rs6971 polymorphism of the TSPO gene. Only high-affinity binders (HABs) of [^11^C]PBR28 were included (see Table 1 for main sociodemographic features).

**Table 1:** Sociodemographic data at Screening Visit.

Subject	Age (years)	Ethnicity	BMI (kg/m^2^)
1	28	Black-African	20.38
2	25	Black-African	23.12
3	33	Asian-Filippino	24.54
4	43	Black-African	24.68
5	29	White-British	23.62
6	26	Black-Carribean	25.62
7	25	Mixed	22.24
Mean ± SD	29.85 ± 6.44	—	23.46 ± 1.75

### Study design

**Fig. 1:**
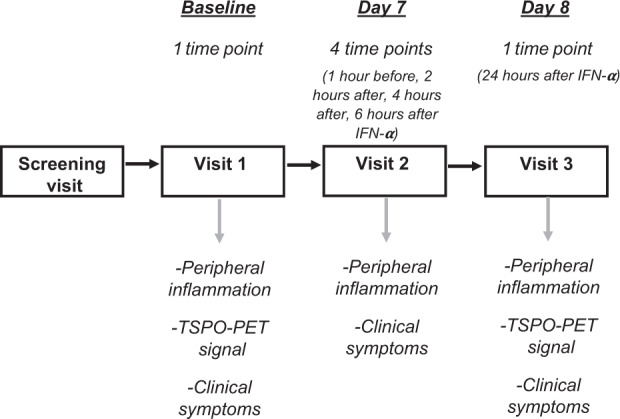
Study design. After a screening visit to assess their eligibility, participants attended three more visits. TSPO-PET signal was collected at 2 timepoints (before and after the immune challenge); data on peripheral inflammation and clinical symptoms were collected at 6 timepoints (2 before and 4 after the immune challenge).

Each participant was assessed four times: at a Screening Visit, to evaluate their eligibility; at Visit 1, when we collected the baseline venous blood sample to measure C-reactive protein (CRP) as a peripheral inflammatory marker, and participants had their first (baseline) PET scan; at Visit 2, when IFN-α was administered, and serum peripheral biomarkers (CRP and cytokines) and clinical symptoms were measured 1 h before, and at 2, 4, and 6 h after the injection; and at Visit 3, 24 h after the administration of IFN-α, when serum peripheral biomarkers (CRP and cytokines) and clinical symptoms were again assessed, and participants had their second PET scan (see Fig. 1 for a summary of the study design).

The two [^11^C]PBR28 PET scans lasted 90 min each, with 7 or 8 days between the 2 scans for most patients (that is, Visit 1 and Visit 3), although two participants had the second scans 1 and 6 months after the first, but always 24 h after the IFN-α injection (that is, Visit 3 was always 24 h after Visit 2) (Fig. 1). To minimise intra and intersubject variability, and limiting the effects of circadian rhythm on TSPO density^33,34^, all scans were conducted between 13:00 and 15:30, and within each individual both scans were conducted at exactly the same time (Table 1, Supplemental Material). All subjects underwent high-resolution T1 magnetic resonance imaging (MRI), before IFN-α administration, in a Siemens Tim Trio 3 T scanner (Siemens healthcare, Erlangen, Germany); these structural MRI images were co-registered with the PET imaging to identify the anatomical regions of interest. All experimental variables related to PET imaging acquisition are described in Supplemental Materials.

### IFN-α administration

At visit 2, participants received a subcutaneous injection of IFN-α 2a (Roferon-A 3 million IU/0.5 ml solution for injection). We monitored vital signs (heart rate, blood pressure, temperature) and the occurrence of adverse effects every hour for 8 h after the injection. Participants were allowed to take 1 gr of paracetamol, once or twice, if sickness symptoms were difficult to tolerate, and six out of the seven participants did so.

### PET imaging

an initial low-dose computer tomography (CT) scan was acquired for attenuation and scatter correction using Siemens Biograph™ True Point™ PET/CT scanner (Siemens Medical Systems, Germany). Subjects then received a bolus injection of [^11^C]PBR28 (target dose ∼300 Mbq) followed by a 90-min PET emission scan. PET data were acquired in 3D mode and binned into 26 frames (durations: 8 × 15 s, 3 × 1 min, 5 × 2 min, 5 × 5 min, 5 × 10 min). Images were reconstructed using filtered back projection and corrected for attenuation and scatter. Radiopharmaceutical preparation acquisition protocol were consistent with previous studies^35–37^.

In parallel to the PET acquisition, arterial blood was sampled from the radial artery using a combined automatic and manual approach. A continuous sampling system (ABSS Allogg, Mariefred, Sweden) was used to measure whole blood activity for the first 15 min of each scan at the rate of one sample per second. Discrete blood samples were manually taken at 5, 10, 15, 20, 25, 30, 40, 50, 60, 70, 80, and 90 min, centrifuged and used to determine the plasma over blood activity ratio (POB). Samples at 5, 10, and 15 min were used to calibrate the two sampling modalities. Samples taken at 5, 10, 20, 30, 50, 70, and 90 min were also analysed using radio-high performance liquid chromatography (HPLC) to calculate the plasma fraction of ligand free of metabolites (PPf). Radiometabolite analysis of [^11^C]PBR28 in the blood was done as described previously^35^. Ligand free-plasma fraction (fp), or the portion of [^11^C]PBR28 unbound to plasma proteins, was determined for all scans using ultrafiltration-based method as previously described^6^.

### Imaging analysis

Structural MRI images were used for grey/white matter segmentation and region of interest (ROI) definition. A neuroanatomical atlas was co-registered on each subject’s MRI scan and PET image using a combination of Statistical Parametric Mapping 8 (http://www.fil.ion.ucl.ac.uk/spm) and FSL (http://www.fsl.fmrib.ox.ac.uk/fsl) functions, implemented in MIAKAT™ (http://www.imanova.co.uk). ROIs included occipital lobe, temporal lobe, frontal lobe, parietal lobe, insular cortex, cingulate cortex, amygdala, hippocampus, thalamus, striatum, and cerebellum. All PET images were corrected for head movement using non-attenuation-corrected images as they include greater scalp signal, which improves realignment compared to attenuation-corrected images. Frames were realigned to a single “reference” space identified by the PET frame with the highest activity. The transformation parameters were then applied to the corresponding attenuation-corrected PET frames to create a movement-corrected dynamic image for analysis. Regional tissue-time activity curves (TACs) were obtained by sampling the image with the co-registered atlas.

### Arterial blood data processing

Processing of blood samples was performed consistently with previous studies^35^. Both POB and PPf were fitted with an extended Hill model^38^ that provided the best data description^39^. Whole blood TACs were fitted using a variation of Feng’s model that consists in a straight line to the arterial input function peak followed by a tri-exponential decay^40^. For each scan, the difference between ligand arrival time in the brain and arterial sampling site was estimated by shifting blood curves 0–20 s (both parent and whole blood TACs), fitting the whole brain TAC (using exponential spectral analysis to avoid dependency on a particular compartmental model), and selecting the delay that produced the smallest weighted residual sum of squares.

### Kinetic analysis

Quantification of [^11^C]PBR28 tissue distribution was performed using both the standard 2TCM and the 2TCM-1K with total distribution volume (Vt) as main parameter of interest^41^. The two models were then used to assess Vt changes before and after IFN-α (%ΔVt), as done in a previous study^42^.

### Biomarkers of peripheral inflammation

At Visit 1 (baseline), a blood sample was collected for the first CRP analysis at the time of the first PET scan. At Visit 2, blood samples were taken 1 h before IFN-α injection, and at 2, 4, and 6 h after IFN-α injection, and then, at Visit 3, at 24 h after IFN-α injection, to measure CRP and other immune biomarkers, based on previous work by Cassidy et al.^43^ (Fig. 1). Serum high sensitivity C-reactive protein (hsCRP) was assayed on the Siemens Advia 2400 Chemistry analyser (Siemens Healthcare Diagnostics, Frimley, UK)^44^. Serum cytokines were measured using Meso Scale Discovery (MSD) V-PLEX sandwich immunoassays^45,46^, and plates read on an MSD QuickPlex SQ 120, as in a previous study conducted in our laboratory^47,48^. MSD Pro-inflammatory Panel 1 (human) kit was used for the measurement of IFN-γ, IL-1β, IL-2, IL-4, IL-6, IL-8, IL-10, IL-12p70, IL-13, and TNF-α, and a custom Cytokine Panel 1 (human) kit was used for the measurement of IL-7, IL-17 A and vascular endothelial growth factor VEGF-A. The inter-assay coefficient of variations was <10%. The results were analysed using MSD DISCOVERY WORKBENCH analysis software. Finally, levels of serum S100B protein were measured in serum as a marker of blood–brain barrier (BBB) permeability^49,50^, using a S100B kit distributed by Diasorin, Charles House, Toutley Road, Wokingham, Berkshire, run on the Liaison XL chemiluminescence analyser^51,52^.

We measured levels of tryptophan, kynurenine and kynurenine pathway metabolites, 3-hydroxykynurenine and kynurenic acid^53,54^ using automated online solid-phase extraction HPLC-tandem mass spectrometry (see Supplemental Material).

### Clinical symptoms

The Mini International Neuropsychiatric Interview (MINI) was used at Screening Visit to diagnose a previous history of psychiatric disorders^55^. At all timepoints, from Visit 1 to Visit 3, the Profile of Mood States (POMS-2) and the State Anxiety Inventory (STAI-S) were administered to assess how participants were feeling “right now”. These are self-administered tools, which allow to easily test-retest affect states and sickness symptoms^56,57^. In particular, POMS and STAI-S have already been used in studies assessing behavioural symptoms following IFN-α^58^ and LPS injection^59^.

### Statistical analysis

We performed two-tailed, paired *t*-tests with *p* < 0.05 to investigate differences in [^11^C]PBR28 signal between baseline and IFN-α challenge (~24 h after the injection). Vt values showed a normal distribution in both 2TCM and 2TCM-K1 models, before and after fp correction (all *p* > 0.5 with the Shapiro-Wilk test). Changes in cytokines and clinical measures were analysed using repeated measures ANOVA followed by paired *t*-test comparing baseline values (1 h before IFN-α) with those at 2, 4, 6, and 24 h. Correlation analyses were performed to test the association between changes in Vt and changes in cytokines levels and in clinical symptoms scores. While clinical symptoms scores were normally distributed, cytokines values were not, so we applied a logarithmic transformation. All data are presented as mean and standard errors of the mean.

## Results

### Measured [^11^C]PBR28 PET uptake is influenced by inflammation-induced changes in the free-plasma fraction of the radioligand, and there are no IFN-α effects once adjusted for this

**Fig. 2:**
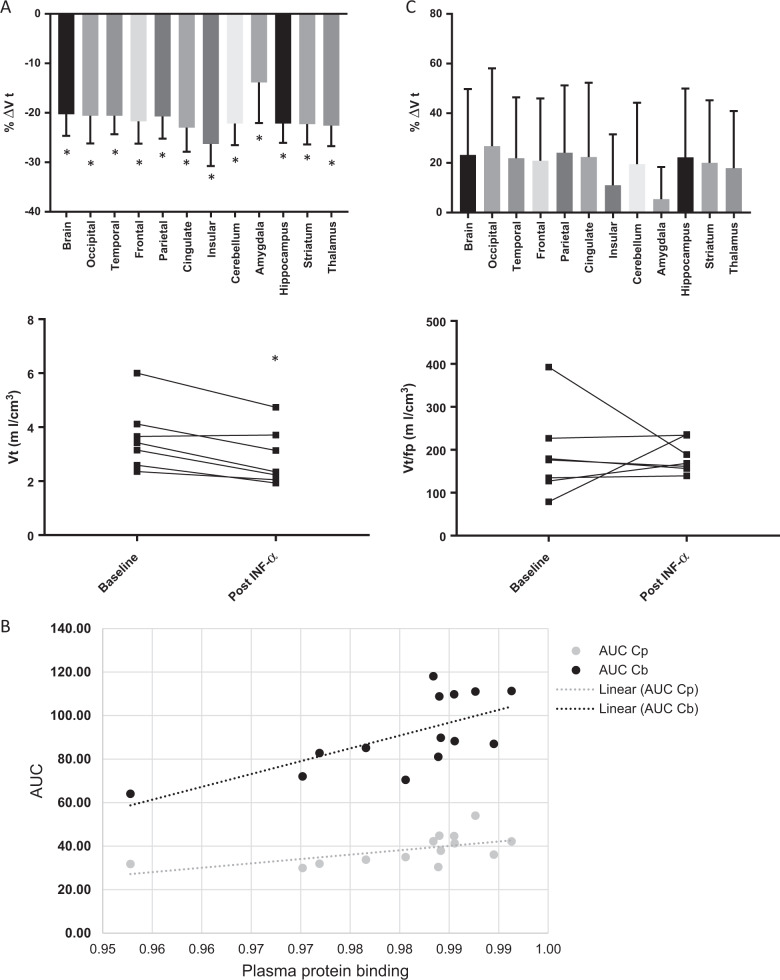
IFN-α administration effect on [^11^C]PBR28 binding (Vt) from baseline in healthy control subjects (*n* = 7). Results before (**a**) and after (**c**) fp correction are shown as regional percent increase in Vt (ΔVt) averaged across subjects (top images error bars are SEM) and Vt _change_ for each subject averaged across regions (bottom images). IFN-α administration decreased [^11^C]PBR28 Vt across the brain, but after correction for radioligand free-plasma fraction there were no longer any changes. **b** Peak activities and area under the curve (AUC) for both plasma (Cp) and whole blood (Cb) showed a significant increase in the second scan and positively correlated with plasma protein binding.

The administration of IFN-α led to a generalized reduction of [^11^C]PBR28 PET uptake across the entire brain in all subjects but one (Fig. 2a, lower part). Between baseline and 24 h after the challenge, the 2TCM model showed a whole brain mean signal reduction (%ΔVt mean ± SE) of −20 ± 4% (paired *t*-test t = −4.18, *p* = 0.01), with only one participant showing a 2% increase. The other regions of interest showed a similar pattern of reduction, ranging from −14% for the Amygdala, to −26% for the Insular cortex (Fig. 2a, upper part).

Interestingly, 77% of this change in Vt between the two scans was caused by a change in tracer blood-to-tissue transport (K1), suggesting a reduced availability of ligand blood flow to the brain. In order to examine whether this reduced availability might be related to the ligand binding to plasma proteins, we further analyzed the ligand free-plasma fraction (fp) and its effect on the arterial blood and plasma [^11^C]PBR28 activity. Indeed, the fp decreased significantly in the second scan, (%Δfp = −18 ± 16%, mixed effects modelling z = −2, *p* = 0.045). Similarly, arterial whole blood (Cb) and plasma (Cp) [^11^C]PBR28 radioactivity were significantly affected by IFN-α administration, compared to baseline conditions, with a pattern indicating a reduction in the ligand available to bind to TSPO in the brain. Peak activities and area under the curve (AUC) for both Cp and Cb TACs significantly increased in the second scan, with relative differences of 30 ± 13% for peak Cp (paired *t*-test t_6_ = −2.63, *p* = 0.04), 25 ± 4% for AUC Cp (paired *t*-test t_6_ = −5.11, *p* = 0.002), 14 ± 8% for peak Cb (paired t-test t_6_ = −1.91, *p* = 0.11), and 34 ± 6% for AUC Cb (paired *t*-test t_6_ = −6.23 *p* = 0.004). Moreover, a positive correlation was found between the peripheral plasma binding (1-fp) and both AUC Cp (Pearson’s r = 0.61, *p* = 0.02) and AUC Cb (Pearson’s r = 0.64, *p* = 0.01) (Fig. 2b). These associations were consistent with increased retention of the ligand in plasma to the increased peripheral inflammatory proteins induced by IFN-α.

Considering the increased plasma binding of the ligand resulting from arterial blood analyses, we corrected results for fp. Interestingly, after this correction, Vt paired *t*-test between the 2 scans dramatically changed and was no longer significant, with four subjects showing an increase in Vt (ranging from 3 to 198%) and three subjects showing a decrease (ranging from −52 to −9%) (Fig. 2c, lower part), averaging 23 ± 31% (t = 0.1, *p* = 0.91). Similar results were present when analyzing specific ROIs (Fig. 2c, upper part).

Results did not change after we repeated all the analyses using the endothelial model 2TCM-1K, again showing a significant decrease using the unadjusted mean whole brain %ΔVt (−28 ± 19%, t = −3.06, *p* = 0.02) and no differences in %ΔVt after fp correction (7 ± 54%, t = 0.59, *p* = 0.57) (Fig. 2a–c).

### IFN-α increases peripheral biomarkers of inflammation

**Fig. 3:**
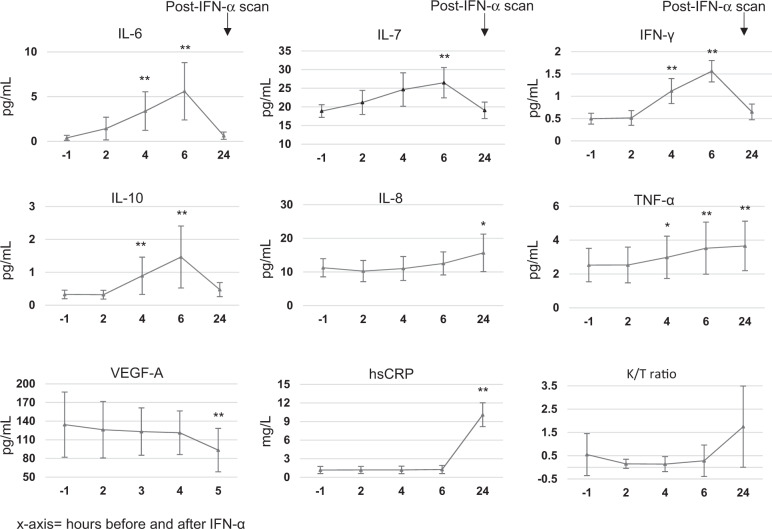
Peripheral response to IFN-α. Peripheral cytokines levels increased after 4–6 h after the IFN-α challenge; hsCrp levels raised at 24 h after IFN-α; K/T ratio increased at 24 h after IFN-α. Data are presented as mean and standard errors of the mean.

IFN-α administration resulted in significant changes in serum biomarkers at 4–24 h after the challenge. Compared with 1 h before IFN-α, repeated measures ANOVA was significant for hsCRP (F_1.2,7.1_ = 44.3, *p* < 0.001), IL-6 (F_4,24_ = 29.70, *p* < 0.001), IL-7 (F_4,24_ = 6.49, *p* = 0.001), IL-8 (F_2.5,15.3_ = 9.11, *p* < 0.001), IL-10 (F_1.3,7.9_ = 64.62, *p* < 0.001), IFN-γ (F_1.6,10.0_ = 32.27, *p* < 0.001), TNF-α (F_4,24_ = 21.10, *p* < 0.001), and VEGF-A (F_4,24_ = 7.71, *p* < 0.001), with significant increases (using LSD pairways comparisons) at both 4 and 6 h for all biomarkers, except hsCRP and IL-8 which increased at 24 h only, IL-7 which increased at 6 h only, and VEGF-A which decreased at 24 h (Fig. 3). Of note is also that TNF-α was still increased at 24 h. Kynurenine/Tryptophan (K/T) ratio also showed an increase at 24 h, but this did not reach statistical significance (86 ± 59.5 folds, F_4,20_ = 1.94, *p* = 0.1) (Fig. 3). S100B protein did not change significantly (change at 24 h: 35 ± 60%, F_1.1,6.7_ = 3.4, *p* = 0.1).

Values of hsCRP measured at the time of the baseline and post-interferon PET scan correlated with the ligand protein binding (1-fp) (Pearson’s r = 0.57, *p* = 0.03) and were inversely associated with K1 values estimates (Pearson’s r = −0.75, *p* = 0.002), indicating that higher peripheral inflammation was associated with increased binding of the ligand to plasma protein and decreased availability of the ligand for entry into the brain.

### IFN-α induces transient mood changes and sickness symptoms

**Fig. 4:**
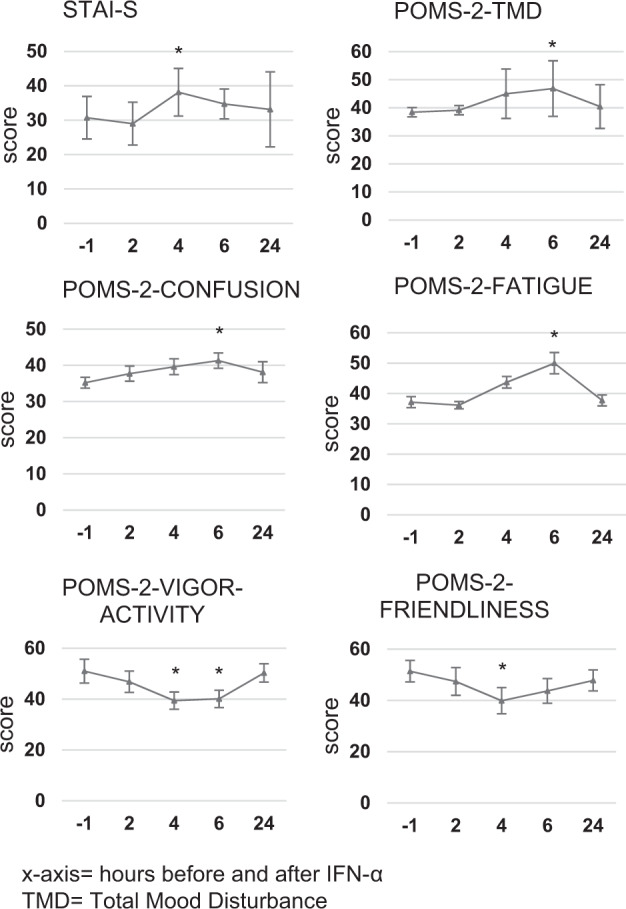
IFN*-*α administration effect on clinical measures over time. POMS-2 and STAI-S scores increased significantly between 4 and 6 h after the challenge. Data are presented as mean and standard errors of the mean.

We found increased sickness symptoms at 4–6 h after the challenge (Fig. 4), as shown by an increase in POMS-2 Total Mood Disturbance (TMD) (F_4,24_ = 4.6, *p* = 0.006), POMS-2 Confusion-bewilderment (F_4,24_ = 3.12, *p* = 0.03) and POMS-2 Fatigue-Inertia (F_1.2,7.2_ = 7.71, *p* = 0.02) at 6 h, and a reduction in POMS-2 Vigor-Activity (F_4,24_ = 5.86, *p* = 0.02) and POMS-2 Friendliness (F_4,24_ = 3.80, *p* = 0.02) at 4 h. Finally, the STAI-S scores peaked at 4 h after IFN-α (F_4,24_ = 2.94, *p* = 0.04). Delta hsCRP (at 24 h) was positively correlated with delta POMS TMD score (at 6 h) (Pearson’s r = 0.88, *p* = 0.009).

### IFN-α increases body temperature, blood pressure, and heart rate

Vital signs were closely monitored after IFN-α administration. From 1 h before the injection to 8 h after, we detected significant increases in body temperature (+1.1 °C, from 36,3 °C to 37,4 °C, t = −6.10, *p* = 0.001) and heart rate (+24 bpm, from 65 bpm to 89 bpm, t = −3.1, *p* = 0.02). Average but not statistically significant increases were found also in systolic blood pressure (+16 mmHg, from 123 mmHg to 139 mmHg) and diastolic blood pressure (+3 mmHg, from 63 mmHg to 66 mmHg).

## Discussion

Here we investigate the association between peripheral and central inflammation in healthy humans, using the immune challenge IFN-α. We show that IFN-α induces peripheral inflammation that is comparable, and sometimes more intense, than that described in depression, in association with acute mood changes. However, we cannot measure a neuroinflammatory response with TSPO-PET imaging. Our thorough methodology addresses all limitations affecting second generation high-affinity tracers: we only select high-affinity binders (HABs); we use both 2TCM and 2TCMK1 models to correct for endothelial binding; and we normalize results for fp, that is, for the amount of ligand that is able to enter the brain because it is free from binding to plasma proteins.

Overall, this study confirms that brain TSPO binding is strongly affected by the free-plasma fraction artefact^32^. The evidence that Vt changes are mainly explained by blood flow (K1) changes, and that the initial Vt decrease disappears after fp correction, indicates that the signal differences between the two PET scans are mainly driven by changes in the availability of free ligand in the plasma after the immune challenge. This is further supported by the positive correlation between inflammation (hsCRP levels) and ligand protein binding, and the negative correlation between hsCRP levels and K1. Our data thus support the notion that peripheral inflammation, here occurring with the IFN-α injection, induces an increase in the ligand’s peripheral plasma binding and thus results in a lower proportion of ligand available for TSPO binding in the brain. This important methodological limitation suggests that the pathophysiological implications of altered brain TSPO-PET signal are difficult to interpret, thus limiting its usefulness as a sensitive marker of neuroinflammation and microglial activation. Of course, our findings are in apparent contrast with the aforementioned studies, which found increased brain TSPO-PET signal in healthy humans after LPS injection, or in patients with depression, a condition associated with increased inflammation. However, many mechanisms can explain this potential discrepancy.

In the aforementioned study^23^, LPS administration in eight healthy males induced much stronger and faster peripheral pro-inflammatory responses compared with IFN-α in our study, partly because LPS acts immediately on toll-like receptors that directly activate the NK-FB pathway^14^. IFN-α, instead, acts through several intermediate steps, including the Jack/STAT/ISGF3 pathway^60^, with the NK-FB pathway as an alternative route of action^61^. For example, IL-6 levels were ~100 pg/ml at 4 h in the LPS study (having reached an average of 200 pg/ml at previous timepoints), while IL-6 levels average around 6 pg/ml at 4 h in our study. Consistent with this, the LPS in that study induced much stronger physiological responses compared with IFN-α in our study, that is, twice the increase in body temperature, and ~+50% in the increase in heart rate. Moreover, these changes happened already at 3 h from the challenge, showing a quicker mechanism of action compared with IFN-α. Finally, and most relevant for the potential discrepancy with our findings, the LPS study did not apply the fp correction, so we have no information on the relationship between acute LPS-induced inflammation, free ligand in the plasma, and brain TSPO signal. Incidentally, this same limitation also applies to the two studies using LPS in non-human primates^21,22^.

The second finding in apparent contrast with our results is the presence of increased TSPO binding in patients with depression, as shown by some of the studies conducted so far. However, it is of note that the two studies which used [^11^C]PBR28 *and* corrected for fp effect, like we do, found no difference^6^, or a borderline-significant increase, in TSPO-PET signal^11^. The other studies that found increased TSPO-PET signal^7–10,18^ used different, first and second generation radioligands, and did not correct for fp, and so they did not take into consideration the ligand binding to plasma proteins. Moreover, of the studies reporting an increased TSPO-PET signal, only two examined depressed patients with increased inflammation^8,10^, and TSPO-PET signal in depressed patients did not correlate with peripheral inflammation.

It is interesting to note that the levels of peripheral inflammation in our study do indeed resemble those present in patients with major depression. For example, the Hedges effect size of IL-6 and TNF-α differences from baseline to 24 h after IFN-α are 0.48 and 0.66, respectively, which are very close to the difference between controls and patients with depression for these two cytokines, as shown by Hedges g = 0.62 and 0.68, respectively^62^. Values of hsCRP at 24 h are higher in our study compared with average depressed patients’ values, as we obtain a mean of around 10 mg/L and Hedges’ g = 1.69 compared with baseline, vs values in most depressed patients between 1 and 3 mg/L^13^ and Cohen’s d = 0.47 for the difference between controls and depressed patients^63^. However, values reached in our study still have clinical relevance for mental health, as one previous study has found that CRP levels above 10 mg/L are associated with a high risk of developing future depression^64^. Thus, the immune challenge with acute IFN-α can be used to examine the neural and immunological regulatory response to an immune perturbation that is within the same magnitude of that described in depression, as opposed to, for example, the much more intense activation of inflammatory processes induced by LPS in the aforementioned study^23^. Further discussion of our immune findings, including the reduction of VEGF-A, is in the Supplementary Material.

Of course, brain TSPO-PET signal is markedly upregulated in clinical conditions that have neuroinflammation at their core, such as Huntington’s disease or multiple sclerosis. Indeed, positive correlations between levels of pro-inflammatory cytokines and brain TSPO-PET signal have been found in neurological conditions associated with genuine neuroinflammatory processes^65^, but not in studies of psychiatric patients^66,67^. Thus, beyond the different technical approaches in the analyses of the PET data, the variability in the results of TSPO-PET studies in psychiatric patients (or in studies that, as ours, mimic the levels of peripheral inflammation described in psychiatric patients), may simply reflect a true lack of microglial activation. Indeed, Notter and colleagues^67^ have recently highlighted that the brain expression of inflammation-related genes, the microglial phenotypes, the presence of reactive gliosis, and the levels of cerebrospinal fluid (CSF) cytokines, are all very different in true neuroimmunological disorders, such as multiple sclerosis, compared with psychiatric disorders. Thus, PET radiotracers targeting alternative markers of immune response might be needed to capture inflammatory processes in the brains of patients with psychiatric disorders^68^, together with CSF analyses, as recently done by Felger and colleagues^69^ and other studies in depressed patients^4^. Moreover, TSPO signal can be driven by factors other than microglial activation^70^, such as recruitment of peripheral monocytes into the parenchyma, adherence of circulating leucocytes to the vascular endothelium, and the expression of TSPO in various brain cells, including microglia, astrocytes, vascular endothelial cells and neurons. Unfortunately, the lack of cellular specificity is often neglected when interpreting PET studies, as well the potential changes in blood–brain barrier permeability and the need to correct for the endothelial component of TSPO signal (as we do in our study). Nevertheless, as our challenge is acute, we cannot exclude the possibility that some depressed patients do have increased brain TSPO-PET signal because of microglial activation, as a consequence of increased inflammation lasting months or even years before the study, rather than just 24 h. In fact, long-lasting inflammation in depression may be driven either by early risk factors, such as childhood maltreatment or antenatal depression^71–73^, or present during previous relapses or exacerbations, even if not measurable on the day of the scan.

This study has some limitations. First, it is possible that the timing of the second PET scan might not have been ideal to detect neuroinflammation. Although we had based our decision to do the second scan at 24 h on preclinical studies^29^, we find that significant sickness symptoms were present at around 4–6 h after the challenge, together with the peak in IL-6, IL-10, and IFN-γ, so we cannot exclude that an increased brain TSPO binding could have been measured at that timepoint. It is also possible that a regulatory anti-inflammatory response could have started at 24 h, and this could have confounded the results. However, some other inflammatory markers relevant to depression were either increased only at 24 h (hsCRP, IL-8) or increased at all timepoints, including 24 h (TNF-α), indicating that systemic inflammatory processes were still present at the time of the second scan. Our research was also limited by the small sample size. However, PET studies are extremely invasive, challenging and expensive, and studies with two repeated PET scans at such a short distance tend to have numbers in the range of 6–8 subjects, like in the aforementioned study in healthy humans with LPS, which had eight subjects^23^.

## Conclusion

To our knowledge, this is the first study assessing central inflammatory responses to IFN-α in healthy humans by using PET together with an assessment of peripheral inflammatory biomarkers. While the ability of IFN-α to induce acute inflammatory responses and mood changes highlights its potential as an immune model of depression for future studies in healthy humans, we find no evidence of a putative neuroinflammatory response, and we unequivocally demonstrate that brain TSPO-PET signal measurement is confounded by the inflammation-induced changes in [^11^C]PBR28 free ligand in the plasma. In conclusion, there is an urgent call for new targets of microglial activation that could also better clarify the role of TSPO in measuring neuroinflammation, especially if coupled with CSF analysis.

## Supplementary information

Supplemental Material

Supplementary Table S1
