# Supplementary material for: PET imaging shows no changes in TSPO brain density after IFN-α immune challenge in healthy human volunteers
Source: Transl Psychiatry. 2020 Mar 9;10:89. doi: 10.1038/s41398-020-0768-z (PMC7063038; doi:10.1038/s41398-020-0768-z)
Supplement: Supplementary file 1 — Supplemental Material [file 41398_2020_768_MOESM1_ESM.docx]

**Supplemental Materials**

**Methods**

There were no significant differences in the following variables between baseline and post-IFN-α scans: participants average weight (73.2 ± 5.2 kg vs. 73.6 ± 6.1 kg), radioligand dose (336.9±14.8 vs. 344.5±15.9 MBq), injected Mass (3.9±2.2 and 4.1±2.2 ug), specific activity (36.2±14.1 and 36.4± 15.4 GBq/umol), total motion (13.56±6.6 and 13.21± 2.9 mm), as confirmed by all paired t-tests p > 0.05 (Table S1).

**Supplementary Table S1 around here**

Kynurenine pathway metabolites measurement: Blood samples were collected using 3 ml VACUETTE® plasma separation, sodium heparin tubes. We used a high-through put on-line solid-phase extraction-liquid. Plasma was pre-purified by automated on-line solid-phase extraction, with a chromatographic–tandem mass spectrometric (XLC–MS/MS) method using strong cation exchange (PRS, propylsulphonic) cartridges. Chromatographic separation of the analytes and deuterated analogues occurred by C18 reversed phase chromatography. Mass spectrometric detection was performed in the multiple reaction-monitoring mode using a quadrupole tandem mass spectrometer with positive electrospray ionization (see (1)).

**Discussion**

*IFN-α induced changes in IL-7 and VEGF-A levels*

Besides the increases in cytokines that have been repeatedly associated with sickness symptoms (such as IL-6, IL8, IL-10, IFN-γ ,TNF-α), we also find an increase in IL-7 levels, which probably reflects the ability of IFN-α to stimulate growth factors involved in lymphoid cell proliferation, as part of its anti-viral effects (2). Our data also confirm the ability of IFN-α to reduce VEGF-A expression (3) (4). As VEGF-A is involved in vasculogenesis and angiogenesis, this confirms IFN-α efficacy in cancer. However, VEGF-A also exerts a vascular protective and anti-inflammatory effect, mediated by Nitric Oxide synthesis, which inhibits leukocytes rolling adhesion to the vascular endothelium (5). It is also known as a neurotrophic factor, thought to exert neuroprotection (6), stimulate axonal outgrowth, enhance cell survival (7) and influence synaptic plasticity (8). In patients with depression, as highlighted by a work from our group (9), studies have found both increased and decreased VEGF mRNA, as well as no difference with healthy controls. A possible interpretation for the elevated VEGF levels in depression appears to be the potential neuroprotective effect against stress, whereas decreased levels of VEGF are observed in treatment-resistant depressed patients whose brains are less able to undergo neurogenesis processes (10).

1. de Jong WH, Smit R, Bakker SJ, de Vries EG, Kema IP. Plasma tryptophan, kynurenine and 3-hydroxykynurenine measurement using automated on-line solid-phase extraction HPLC-tandem mass spectrometry. J Chromatogr B Analyt Technol Biomed Life Sci. 2009;877(7):603-9.

2. Hou L, Jie Z, Liang Y, Desai M, Soong L, Sun J. Type 1 interferon-induced IL-7 maintains CD8+ T-cell responses and homeostasis by suppressing PD-1 expression in viral hepatitis. Cellular & molecular immunology. 2015;12(2):213-21.

3. von Marschall Z, Scholz A, Cramer T, Schafer G, Schirner M, Oberg K, et al. Effects of interferon alpha on vascular endothelial growth factor gene transcription and tumor angiogenesis. J Natl Cancer Inst. 2003;95(6):437-48.

4. Rosewicz S, Detjen K, Scholz A, von Marschall Z. Interferon-alpha: regulatory effects on cell cycle and angiogenesis. Neuroendocrinology. 2004;80 Suppl 1:85-93.

5. Zachary I. Signaling mechanisms mediating vascular protective actions of vascular endothelial growth factor. Am J Physiol Cell Physiol. 2001;280(6):C1375-86.

6. Storkebaum E, Lambrechts D, Carmeliet P. VEGF: once regarded as a specific angiogenic factor, now implicated in neuroprotection. Bioessays. 2004;26(9):943-54.

7. Sondell M, Lundborg G, Kanje M. Vascular endothelial growth factor stimulates Schwann cell invasion and neovascularization of acellular nerve grafts. Brain Res. 1999;846(2):219-28.

8. Licht T, Goshen I, Avital A, Kreisel T, Zubedat S, Eavri R, et al. Reversible modulations of neuronal plasticity by VEGF. Proc Natl Acad Sci U S A. 2011;108(12):5081-6.

9. Nikkheslat N, Zunszain PA, Horowitz MA, Barbosa IG, Parker JA, Myint AM, et al. Insufficient glucocorticoid signaling and elevated inflammation in coronary heart disease patients with comorbid depression. Brain Behav Immun. 2015;48:8-18.

10. Clark-Raymond A, Halaris A. VEGF and depression: a comprehensive assessment of clinical data. J Psychiatr Res. 2013;47(8):1080-7.

**Tables and figures caption**

**Supplementary Table S1** PET experimental variables
