# Supplementary material for: PET imaging shows no changes in TSPO brain density after IFN-α immune challenge in healthy human volunteers
Source: Transl Psychiatry. 2020 Mar 9;10:89. doi: 10.1038/s41398-020-0768-z (PMC7063038; doi:10.1038/s41398-020-0768-z)
Supplement: Supplementary file 2 — Supplementary Table S1 [file 41398_2020_768_MOESM2_ESM.docx]

|  | **Subject** | **Scan time** | **Dose *(MBq)*** | **Injected Mass *(ug)*** | **Specific activity *(GBq/umol)*** | **Tot Motion *(mm)*** | **Max InterFrame Motion*(mm)*** | **fp** |
| --- | --- | --- | --- | --- | --- | --- | --- | --- |
| ***Baseline*** | ***1*** | 15:17:54 | 348.95 | 3.03 | 40.16 | 10.08 | 2.03 | 0.05 |
|  | ***2*** | 15:34:03 | 318.99 | 3.69 | 30.09 | 13.14 | 1.26 | 0.02 |
|  | ***3*** | 15:00:14 | 331.30 | 1.97 | 58.69 | 13.19 | 2.00 | 0.02 |
|  | ***4*** | 13:01:23 | 318.41 | 3.40 | 32.64 | 13.74 | 2.40 | 0.01 |
|  | ***5*** | 15:33:48 | 336.93 | 8.79 | 13.35 | 27.73 | 10.95 | 0.01 |
|  | ***6*** | 15:02:09 | 355.57 | 2.70 | 45.85 | 7.41 | 0.87 | 0.02 |
|  | ***7*** | 15:36:48 | 348.24 | 3.70 | 32.78 | 9.66 | 1.60 | 0.03 |
| ***24 hours after IFN-α*** | ***1*** | 15:04:08 | 352.70 | 3.24 | 37.91 | 16.51 | 4.12 | 0.03 |
|  | ***2*** | 15:00:35 | 372.73 | 3.11 | 41.74 | 11.42 | 1.99 | 0.01 |
|  | ***3*** | 15:30:41 | 342.69 | 2.57 | 46.46 | 16.14 | 3.84 | 0.02 |
|  | ***4*** | 13:02:58 | 329.73 | 7.46 | 15.39 | 16.32 | 2.59 | 0.02 |
|  | ***5*** | 15:52:04 | 325.59 | 2.93 | 38.71 | 10.56 | 0.75 | 0.01 |
|  | ***6*** | 15:10:15 | 338.45 | 7.05 | 16.73 | 9.81 | 1.11 | 0.02 |
|  | ***7*** | 15:00:03 | 349.96 | 2.11 | 57.73 | 11.69 | 1.20 | 0.01 |

**Supplementary Table S1** PET experimental variables
